# First Insights on the Karyotype Diversification of the Endemic Malagasy Leaf-Toed Geckos (Squamata: Gekkonidae: *Uroplatus*)

**DOI:** 10.3390/ani12162054

**Published:** 2022-08-12

**Authors:** Marcello Mezzasalma, Elvira Brunelli, Gaetano Odierna, Fabio Maria Guarino

**Affiliations:** 1 Department of Biology, Ecology and Earth Science, University of Calabria, Via P. Bucci 4/B, 87036 Rende, Italy; 2 Department of Biology, University of Naples Federico II, Via Cinthia 26, 80126 Naples, Italy

**Keywords:** evolution, karyotype, NORs, Madagascar, reptiles, sex chromosomes

## Abstract

**Simple Summary:**

The geckos of the genus *Uroplatus* include peculiar endemic species to Madagascar. Even though they have been the subject of several morphological and molecular studies, karyological analyses have been performed only on *U. phantasticus*, leaving the chromosomal diversity of the genus completely unexplored. In this study, we performed a preliminary molecular analysis and a comparative cytogenetic study providing the first karyotype description of eight species of *Uroplatus* and an assessment of their karyological variability. We found chromosome diversity in the species studied in terms of total chromosome number (2n = 34–38), localization of loci of Nucleolar Organizer Regions (NORs) (alternatively on the 2nd, 6th, 10th or 16th pair), heterochromatin composition and occurrence of heteromorphic sex chromosome pairs. Adding our newly generated data to those available from the literature, we show that in the genus *Uroplatus*, as well as in a larger group of phylogenetically related gecko genera, chromosome diversification mainly occurred toward a reduction in the chromosome number by means of chromosome fusions and translocation of NOR-bearing chromosomes. We also hypothesize that the diversification of sex chromosome systems occurred independently in different genera.

**Abstract:**

We provide here the first karyotype description of eight *Uroplatus* species and a characterization of their chromosomal diversity. We performed a molecular taxonomic assessment of several *Uroplatus* samples using the mitochondrial 12S marker and a comparative cytogenetic analysis with standard karyotyping, silver staining (Ag-NOR) and sequential C-banding + Giemsa, +Chromomycin A3 (CMA_3_), +4′,6-diamidino-2-phenylindole (DAPI). We found chromosomal variability in terms of chromosome number (2n = 34–38), heterochromatin composition and number and localization of loci or Nucleolar Organizer Regions (NORs) (alternatively on the 2nd, 6th, 10th or 16th pair). Chromosome morphology is almost constant, with karyotypes composed of acrocentric chromosomes, gradually decreasing in length. C-banding evidenced a general low content of heterochromatin, mostly localized on pericentromeric and telomeric regions. Centromeric bands varied among the species studied, resulting in CMA_3_ positive and DAPI negative or positive to both fluorochromes. We also provide evidence of a first putative heteromorphic sex chromosome system in the genus. In fact, in *U. alluaudi* the 10th pair was highly heteromorphic, with a metacentric, largely heterochromatic W chromosome, which was much bigger than the Z. We propose an evolutionary scenario of chromosome reduction from 2n = 38 to 2n = 34, by means of translocations of microchromosomes on larger chromosomes (often involving the NOR-bearing microchromosomes). Adding our data to those available from the literature, we show that similar processes characterized the evolutionary radiation of a larger gecko clade. Finally, we hypothesize that sex chromosome diversification occurred independently in different genera.

## 1. Introduction

Madagascar is one of the world’s “hottest” biodiversity hotspots and an ideal region to better understand complex evolutionary dynamics [1,2,3]. The Malagasy reptile fauna comprises more than 430 terrestrial endemic squamate species and nine different families (Boidae, Lamprophiidae, Typhlopidae, Agamidae, Chamaeleonidae, Gekkonidae, Gerrhosauridae, Opluridae and Scincidae) [4,5]. Among them, the family Gekkonidae includes 11 genera (*Blaesodactylus* Boettger, 1893, *Ebenavia* (Boettger, 1878), *Geckolepis* Grandidier, 1867, *Gehyra* (Wiegmann, 1834), *Hemidactylus* Oken, 1817, *Lygodactylus* Gray, 1864, *Matoatoa* Nussbaum, Raxworthy & Pronk, 1998, *Paragehyra* Angel, 1929, *Paroedura* Günther, 1879, *Phelsuma* Gray, 1825 and *Uroplatus* Duméril, 1806), with a total of more than 100 species currently described [5].

However, even if recent research started to better define the phylogeny and the taxonomy of many different groups, only a small fraction of species has been studied with cytogenetic methods, despite an increasing evidence that their species diversity is reflected at the karyotype level [6,7,8,9,10,11,12].

This applies also to the geckos of the genus *Uroplatus*, which have been the subject of several morphological and molecular studies (see e.g., [13,14,15,16,17,18,19,20,21,22,23]), but only *U*. *phantasticus* (Boulenger, 1888) has a known karyotype, leaving the chromosome diversity of the genus completely unexplored. Overall, the karyotypes of geckos exhibit a wide variability in terms of the total number of chromosomes, number of uni-armed and bi-armed chromosomes, localization of different chromosome markers and presence or absence of differentiated sex chromosomes [6,8,9,24]. In *U. phantasticus*, the karyotype is composed of 2n = 36, all acrocentric chromosomes, Nucleolar Organizer Regions (NORs) on the second pair and absence of differentiated sex chromosomes [24].

The genus *Uroplatus* currently includes 21, mostly nocturnal, forest-dwelling species, which are overall widespread in Madagascar and surrounding islands (such as Nosy Be), with the exception of the arid southern spiny forest and regions 2400 m asl [15]. The genus also includes several regional endemic and candidate species which are awaiting formal description, highlighting that the species diversity is currently underestimated (e.g., [15,23]).

In this paper we performed a preliminary molecular taxonomic analysis and a comparative cytogenetic study with standard karyotyping, Ag-NOR staining and sequential C-banding on different *Uroplatus* samples from distinct Malagasy areas. We provide the first karyotype description of eight species of the genus and a characterization of their chromosomal diversity. Then, superimposing our newly generated karyological data on available phylogenies [23,25] and comparing our results with available literature data on evolutionary related gecko species [6,8,24,26], we hypothesize that a progressive reduction in the chromosome number (with the formation of metacentric chromosomes and the translocation of NORs) is a common evolutionary trend in different genera.

We also provide a first record of a putative heteromorphic sex chromosome system in the genus and hypothesize that sex chromosome diversification occurred multiple times, independently in the phylogenetically related genera *Paroedura*, *Lygodactylus* and *Christinus* Wells & Wellington, 1983.

## 2. Material and Methods

### 2.1. Sampling

We examined 13 samples of 8 different species of the genus *Uroplatus*. The samples were collected during fieldwork in 1999–2004 by various collaborators and no animal was sampled during the realization of this study. Taxonomic attribution, field number, sex, and origin of all the samples analysed in this study are provided in Table 1.

After capture, animals were injected with a 0.5 mg/mL colchicine solution (0.1 mL/10 g body weight). Tissue samples (intestine, spleen and gonads) were incubated for 30 min in hypotonic solution (KCl 0.075 M + sodium citrate 0.5%, 1:1), fixed and conserved in Carnoy’s solution (methanol and acetic acid, 3:1). The fixed material was preserved at 4 °C and transferred to the laboratory of University of Naples Federico II where it was processed as described below.

### 2.2. Molecular Analysis

DNA was extracted from tissue samples following Sambrook et al. [27]. A fragment of about 450 bp of the mitochondrial 12S rRNA gene was amplified using the primer pair 12Sa 5′-AAACTGGGATTAGATACCCCACTAT−3′ and 12Sb 5′-GAGGGTGAGGGCGGTG-TGT−3′ [28]. This marker was chosen considering its wide use on *Uroplatus* geckos and the number of available sequences in public repositories [13,15,16,17,18,19,20,21,22,23].

PCR was conducted in 25 μL using the following parameters: initial denaturation at 94 °C for 5 min, followed by 36 cycles at 94 °C for 30 s, 55 °C for 30 s, 72 °C for 45 s and a final extension for 7 min at 72 °C. Amplicons were sequenced on an automated sequencer ABI 377 (Applied Biosystems, Foster City, CA, USA) using BigDye Terminator 3.1 (Applied Biosystems, Foster City, CA, USA).

Chromatograms were manually checked and edited using Chromas Lite 2.6.6 (Technelysium Pty Ltd., Brisbane, Australia) and BioEdit 7.2.6.1 [29]. All newly determined sequences were deposited in GenBank (accession numbers: OP094031-OP094043).

For taxonomic attribution, the newly determined sequences were compared with available homologous traits deposited in GenBank which were used in previous phylogenetic and taxonomic studies on the genus *Uroplatus* (see e.g., [13,14,15,16,17,18,19,20,21,22,23].

This preliminary analysis allowed us to perform a taxonomic assessment of the collected samples as reported in Table 1. Given the maximum identity scores between the specimens analysed in this work and deposited sequences of *Uroplatus* used in previous taxonomic studies (99.3–100%), we are confident in the taxonomic attribution provided in Table 1. A notable exception is represented by the specimens GA 328 and GA 329, which are here reported as *U.* prope *guentheri* (Table 1) based on their maximum identity score (96.8%) with a previously deposited homologous sequence of *U. guentheri*, (AN EU596688). Considering the pairwise distance threshold usually used for species identification in squamates for the 12S (3–4%) see e.g., [30,31], it is therefore possible that the samples GA 328 and GA 329 represent an undescribed lineage of *Uroplatus*, but more focused morphological and molecular analyses employing a combination of mitochondrial and nuclear markers should be performed to better assess the taxonomic placement of these samples. This result is not surprising considering the significant number of newly described *Uroplatus* species in the last years and the molecular identification of different undescribed lineages (e.g., [20,23]).

### 2.3. Cytogenetic Analysis

Metaphase plates were obtained from tissues sampled during previous fieldwork (see above) using the air-drying method as described in Mezzasalma et al. [32].

Chromosomes were stained with conventional colorations (5% Giemsa solution at pH 7), silver staining (Ag-NOR) [33], C-banding according to Sumner [34] and sequential C-banding + Chromomycin A3 (CMA_3_), +4′,6-diamidino-2-phenylindole (DAPI). following Mezzasalma et al. [35].

Karyotype reconstruction was performed after scoring at least five plates per sample and chromosomes were classified following Levan et al. [36].

## 3. Results

### Cytogenetic Analysis

Our chromosome analysis showed the occurrence of karyological variability among the studied samples in terms of chromosome number, number and chromosome location of loci of NORs, pattern of heterochromatin and the occurrence of a putative heteromorphic sex chromosome pair.

**Figure 1:**
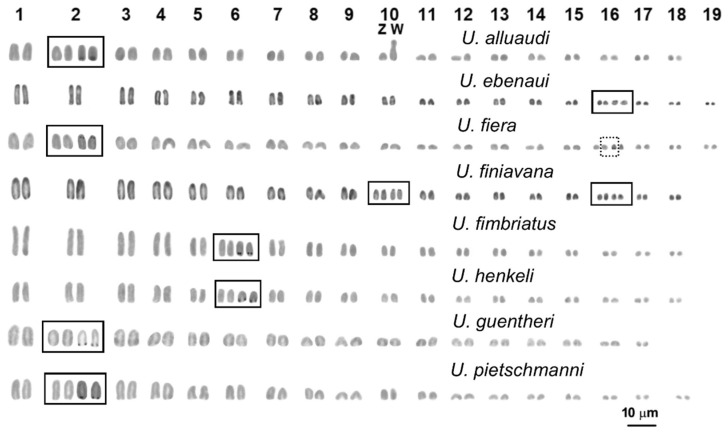
Giemsa stained karyotypes of the studied taxa. Insets include the NOR-bearing pair.

Chromosome number varied from 2n = 34 (in *U*. prope *guentheri*) to 2n = 38 of (in *U. ebenaui* and *U. fiera*). A karyotype of 2n = 36 was the most common condition in the samples studied and shown by five different species (*U. alluaudi*, *U. finiavana*, *U. fimbriatus*, *U. henkeli* and *U. pietschmanni*). The karyotypes of all the analysed specimens were composed of all acrocentric chromosomes, gradually decreasing in length. The only exception was represented by the studied female of *U. alluaudi*, whose karyotype showed a heteromorphic pair (10th pair) including an acrocentric chromosome which was distinctively shorter than a metacentric chromosome. This pair, also in consideration of C-banding results (see below), can be considered as a putative heteromorphic sex chromosome pair with female heterogamety (ZZ/ZW) (Figure 1).

In three species (*U. alluaudi*, *U. guentheri*, and *U. pietschmanni*), loci of NORs were localised in a telomeric position on the chromosomes of the 2nd pair. In two species (*U. fimbriatus* and *U. henkeli*), loci of NORs were in a peritelomeric position on the 6th chromosome pair. In U. *finiavana* NORs were on the chromosomes of the 10th and 16th pair, while in *U*. *ebenaui* NORs were localised on the chromosomes of the 16th pair. Loci NORs were peculiar in *U. fiera*, residing on pericentromeric regions of the chromosomes of the 2nd pair and on one of the chromosomes of the 16th pair (Figure 1).

Given the quantity and quality of metaphase plates, sequential C-banding + CMA_3_ + DAPI + Giemsa was successfully performed only in *U. ebenaui*, *U. finiavana*, *U. pietschmanni* and *U. alluaudi*.

**Figure 2:**
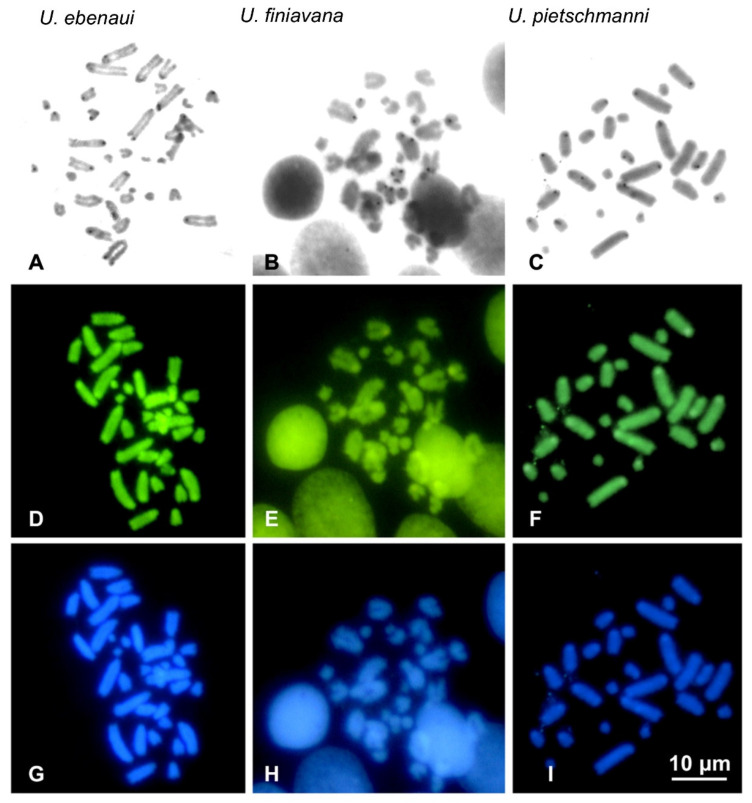
Metaphase plates of *U. ebenaui* (**A**,**D**,**G**), *U. finiavana* (**B**,**E**,**H**) and *U. pietschmanni* (**C**,**F**,**I**) sequentially stained with C-banding + Giemsa (**A**–**D**) + CMA_3_ (**D**–**F**) + DAPI (**G**–**I**).

**Figure 3:**
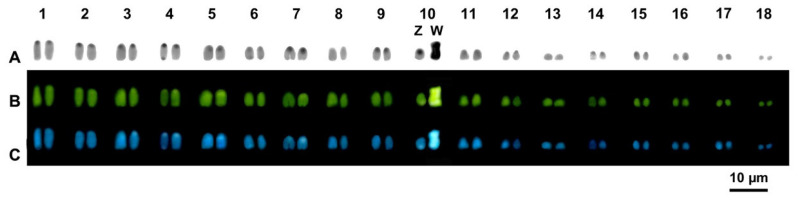
Karyotype of *U. alluaudi* sequentially stained with C-banding + Giemsa (**A**), +CMA_3_ (**B**) and +DAPI (**C**).

C-banding evidenced a low content of heterochromatin in the species studied, with the occurrence of heterochromatic regions on pericentromeric and telomeric regions of almost all chromosomes of all the studied taxa. Nevertheless, although generally barely visible with fluorochromes, centromeric bands varied among different species by being CMA_3_ positive and DAPI negative (in U. *ebenaui* and *U*. *finiavana*) or positive to either CMA_3_ and DAPI (in *U*. *pietschmanni*) (Figure 2). In *U. alluaudi*, C-banding evidenced thin centromeric heterochromatic bands in several chromosome pairs, which were positive to both CMA_3_ and DAPI (Figure 3). Interestingly, the larger (metacentric) chromosome of the heteromorphic pair were completely heterochromatic, positive to both fluorochromes and was therefore identified as a putative W sex chromosome (Figure 3). Because the Z chromosome did not show any distinctive heterochromatic pattern after C-banding, allowing its unambiguously identification among different autosome pairs, the ZW pair was tentatively assigned to the 10th chromosome pair (see Discussion).

## 4. Discussion

Our cytogenetic analysis provided the first karyotype description of eight Malagasy gecko species of *Uroplatus* and represents the first step in describing the karyological variability of the genus, as well as a new contribution to reconstruct chromosomal evolutionary dynamics in a larger clade of leaf-toed geckos.

Overall, we found that the chromosomal diversity in *Uroplatus* mostly encompasses the total chromosome number (from 2n = 34 to 38), a different localization of loci of NORs and the raising of putative heteromorphic sex chromosomes. Chromosome morphology resulted almost invariably acrocentric in the genus with the exception of a large metacentric chromosome found in *U. alluaudi*, here considered as the W sex chromosome (see below).

**Figure 4:**
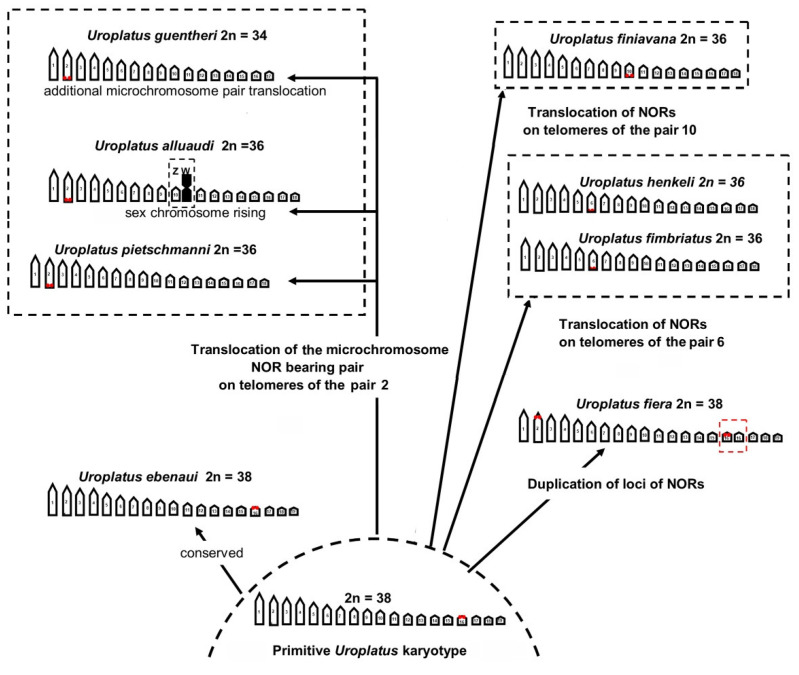
Hypothesized scenario of chromosome diversification in *Uroplatus*.

Taking into account different karyological features which are considered plesiomorphic in squamates (high total number of chromosomes, number of dot-shaped microchromosomes and loci on NORs on the smallest pairs (see e.g., [37,38,39,40,41]), the karyotype of *U. ebenaui* (2n = 38, with NORs on one of the smallest pair) should be considered as a primitive state in *Uroplatus*. From karyotypes with a similar structure, the chromosomal diversification in the genus probably proceeded toward a progressive reduction in the total chromosome number (2n = 36 in *U. phantasticus*, *U. alluaudi*, *U. finiavana*, *U. fimbriatus*, *U. henkeli* and *U. pietschmanni* and 2n = 34 in *U*. prope *guentheri*) ([24] this study) by means of chromosome fusions and translocations of chromosomes of the smallest pairs (Figure 4).

The variability of loci of NORs also plays an important role in the karyotype diversification of the genus *Uroplatus*. In fact, rDNA gene clusters are considered recombination “hotspots” and can induce significant evolutionary changes by means of their translocation among different genomic regions and/or the differential inactivation of different loci [9,40,41]. In *Uroplatus*, the traslocation of NORs probably occurred among different chromosomes, from those of the smallest pairs (16th and 10th pair in *U. finiavana* and *U. ebenaui*) to middle-sized (6th pair in *U. fimbriatus* and *U. henkeli*) and large chromosomes (2nd pair in *U. phantasticus*, *U. alluaudi*, *U. guentheri*, and *U. pietschmanni*) ([24] this study) (Figure 4). The condition displayed by *U. fiera* (NORs on the 2nd pair and an extra, unpaired locus, on one of the chromosomes of the 16th pair), is quite rare in reptiles, but similar configurations have been documented in Lacertidae, Opluridae, Leiocephalidae and Helodermatidae (see e.g., [37,38,39,40,42,43,44,45]).

**Figure 5:**
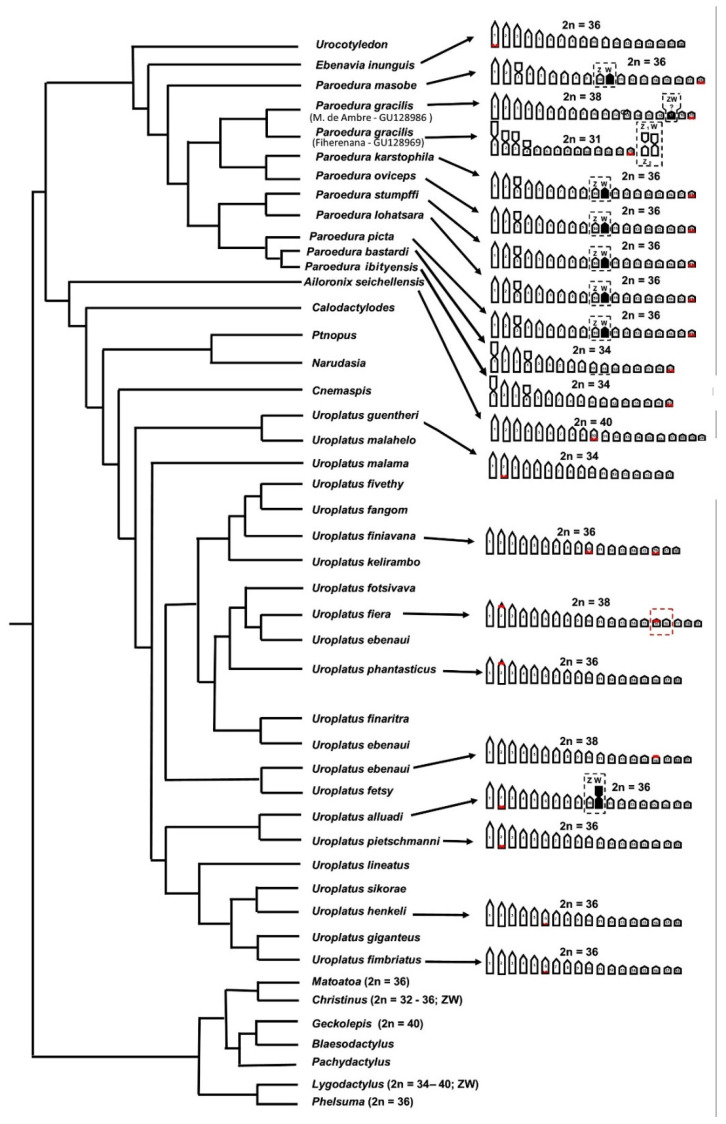
Hypothesized scenario of chromosome diversification in phylogenetically related leaf-toed geckos. Available karyological data from the literature [6,8,9,24,26,44,46,47,48] are superimposed on the phylogenetic tree by Pyron et al. [25], with relationships of the *U. ebenaui* group by Ratsoavina et al. [23].

More in general, the karyotypes of the *Uroplatus* species studied here resemble those of the phylogenetically related Malagasy leaf-toed geckos of the genera *Paroedura*, *Ebenavia*, *Phelsuma*, *Matoatoa* and the Australian genus *Christinus*. To highlight karyological affinities and differences between these phylogenetically related genera we superimposed the haploid karyograms of the studied samples of *Uroplatus*, as well as those available from the literature, to the phylogentic tree by Pyron et al. [25], adding the intrageneric relationships of the *U*. *ebenaui* species group by Ratsoavina et al. [23] (Figure 5).

Similarly, to what has been previously described within *Uroplatus* (see above), *Lygodactylus* [8], *Matoatoa* [44], *Paroedura* and *Christinus* [6,24,26], the whole group seems to be characterized by an overall reduction in the chromosome number and the independent acquisition of derivate chromosome features. In fact, all these genera display a karyotype composed of 2n = 34–42 mostly acrocentric chromosomes, the progressive formation of metacentric chromosomes by means of chromosome fusions in karyotypes with a reduced chromosome number (in e.g., *Lygodactylus*, *Matoatoa*, *Paroedura* and *Christinus*) and/or the translocation of small NOR-bearing chromosomes on larger chromosomes (in e.g., *Uroplatus*, *Matoatoa* and *Ebenavia*) (see Figure 5).

We highlight that this group of geckos provides an example of an early stage of the transition between “symmetrical” (mostly composed by acrocentric chromosomes and without a clear distinction between macro- and microchromosomes) and “asymmetrical” karyotypes (with a high number of biarmed chromosomes and a clear distinction between macro- and microchromosomes) [45], which is hypothesized to represent a major evolutionary trend of the karyological diversification of squamates [39,49].

Another interesting outcome of our cytogenetic analysis is the first record in *Uroplatus* of a putative heteromorphic sex chromosome system (ZW in *U*. *alluaudi*). However, only a single female was studied in this work and more karyological data on males and females of *U. alluaudi* should be gathered in order to confirm this observation. Nevertheless, we highlight that the occurrence of a sex chromosome system is the most robust explanation of the heteromorphic pair found in the female specimen here studied. Notably, the largely heterochromatic W chromosome found in *U. alluaudi* is much bigger than the Z, a condition rarely observed in squamates, e.g., in *Clelia clelia* (Daudin, 1803) and *Phisalixella variabilis* (Boulenger, 1896) [7,50].

Bigger dimensions of the heteromorphic chromosome (Y/W) usually indicate its relatively recent diversification by means of heterochromatin addition and amplification, which is usually followed by the degeneration of the Y/W chromosome, down to the size of a microchromosome [39,51,52,53]. The lack of other heteromorphic sex chromosomes in the other *Uroplatus* species studied so far, also seems to support the relatively recent origin of the heteromorphic pair in *U. ebenaui.*

In phylogenetically related geckos, heteromorphic sex chromosome systems are not a common feature and are known mainly in *Paroedura* (different species with ZW and Z_1_Z_2_W chromosomes), *C. marmoratus* (ZW as the 4th pair) and *L. tuberosus* (ZW as the 1st pair) [6,8,24,26].

Reptiles are a well-known model organism in the study of sex chromosome diversification and include species with temperature dependent sex determination (TSD) and genetic sex determination (GSD) with either male or female heterogamety (see e.g., [39,51,52,53,54,55,56,57,58]).

According to the most supported hypotheses, the process of sex chromosome differentiation begins when a sex determining locus rises in one of the two homomorphic proto-sex chromosomes which are at this step cytogenetically undetectable with standard and banding methods [41,52,53,54,55,56]. The next step of the diversification of the proto-Y/W is the suppression of recombination in the region containing the sex-determining locus by means of an inversion or a progressive heterochromatin addition. This eventually leads to the evolutionary isolation of the Y/W chromosome and to its progressive degeneration. At intermediate and final stages of its diversification, the Y/W chromosome appears dimensionally distinguishable from the X/Z and/or largely heterochromatic [32,41,51,56].

In the gecko clade considered here, sex chromosome diversification seems to have followed different pathways in different genera. Diversification by progressive addition of heterochromatin probably occurred in *L. tuberorus*, six *Paroedura* species and *U*. *alluaudi*. In fact, the W chromosomes of these species show different levels of heterochomatinization; with pseudoautosomal regions (*L*. *tuberosus*) [8], largely heterochromatic but homomorphic (in *Paroedura*) [6,24,57] or heteromorphic and heterochromatic (*U. alluaudi*) (this study).

The alternative model has been proposed for *C. marmoratus*, whose euchromatic, submetacentric W started its diversification from the Z by means of an inversion [26], while the multiple sex chromosome system of *P*. *gracilis* from Fiherenena (2n = 31, with Z_1_Z_1_W) probably originated from an autosome-sex chromosome fusion [24].

It should also be noted that, excluding *Paroedura*, most species and genera (Figure 5) of the gecko clade considered here do not show any heteromorphic or heterochromatic sex chromosomes, suggesting their early diversification stage ([6,8] this study).

In *Paroedura* species with known heteromorphic W chromosomes, the sex chromosome pair is always the 10th, and chromosome painting with Z-specific markers showed pair homology among different species [6,57]. However, Z-specific markers are absent in other species of the genus without differentiated sex chromosomes, as well as in *E. inunguis*, which represents the sister clade to *Paroedura*.

The other species of the clade with known sex chromosome systems show their localization on different pairs. In *U*. *alluaudi* the Z chromosome is not easily distinguishable from different autosome pairs, and we tentatively described it as the 10th pair only based on its dimension. In two other genera, *L*. *tuberosus* shows sex chromosomes on the first pair, while they are on the 4th pair in *C*. *marmoratus* [8,26]. These evidences seem to suggest the independent origin (non-homology) of sex chromosome pairs in these different gecko genera (Figure 5), but more focused analysis with molecular cytogenetics are needed to confirm this hypothesis.

## 5. Conclusions

We provide here the first karyotype description of eight gecko species of the genus *Uroplatus*, which varied in terms of chromosome number (2n = 34–38), localization of loci of NORs (alternatively on the 2nd, 6th, 10th or 16th pair), heterochromatin composition and the occurrence of a putative heteromorphic sex chromosome pair.

Considering the occurrence of chromosome characters which are considered plesiomorphic in squamates, we hypothesise a karyotype of 2n = 38 with NORs on one of the smallest pairs as the primitive condition in *Uroplatus*. Progressive chromosome rearrangements eventually led to karyotypes with a lower chromosome number (2n = 34–36) and NORs on medium or large chromosomes.

Overall, the karyotypes of the *Uroplatus* species studied here resemble those of phylogenetically related leaf-toed geckos, including *Paroedura*, *Ebenavia*, *Phelsuma* and *Matoatoa* and the Australian genus *Christinus*. We show that the whole group is characterized by a tendency toward a reduction in the chromosome number (from 2n = 42 to 2n = 34), the formation of metacentric chromosomes and/or the translocation of NORs on middle-sized or large chromosomes.

We also found a first case of a putative heteromorphic sex chromosome pair in *Uroplatus* (ZW in *U. alluaudi*), with a largely heterochromatic W chromosome which is much bigger than the Z. We discuss similarities and differences of sex chromosome diversification in phylogenetically related taxa (different *Paroedura* species, *L. tuberosus* and *C. marmoratus*), hypothesizing that the rise of non-homologous sex chromosomes occurred independently in different genera.

## Figures and Tables

**Table 1:** Specimens analysed in this study. FN = field number. Max identity = Maximum identity scores with deposited homologous sequences.

Species	FN	Sex	Locality	Max Identity
*U. alluaudi* Mocquard, 1894	GA 476	female	Montagne d’Ambre	100% vs. KF160464
*U. henkeli* Böhme & Ibisch, 1990	GA 477	male	Montagne d’Ambre	99.3% vs. JX205281
*U. henkeli*	GA 1099	male	Montagne d’Ambre	99.3% vs. JX205281
*U. ebenaui* (Boettger, 1879)	FGMV 2205	female	Manongarivo	99.4% vs. JX205278
*U. ebenaui*	GA 1100	female	NA	99.4% vs. JX205278
*U. fiera* Ratsoavina, Ranjanaharisoa, Glaw, Raselimanana, Miralles & Vences, 2015	FGMV 3097	male	Fiherenana region	100% vs. JX205263
*U. fiera*	GA 140	juvenile	Fiherenana region	100% vs. JX205263
*U. finiavana* Ratsoavina, Louis Jr., Crottini, Randrianiaina, Glaw & Vences, 2011	FGMV 3084	male	Montagne d’Ambre	100% vs. MW035835
*U. finiavana*	GA 1100	juvenile	Montagne d’Ambre	100% vs. MW035835
*U. fimbriatus* (Schneider, 1797)	FGMV 2234	male	NA	99.5% vs. AB612276
*U.* prope *guentheri* Mocquard, 1908	GA 328	male	Marofandilia	96.8% vs. EU596688
*U.* prope *guentheri*	GA 329	male	Marofandilia	96.8% vs. EU596688
*U. pietschmanni* Böhle & Schönecker, 2004	FAZC 11627	male	NosyBe	99.7% vs. EU596687

## Data Availability

Newly generated cytogenetic data are available within this manuscript. DNA sequences are available on GenBank (accession numbers: OP094031-OP094043).
